# A High-Throughput Method for Screening Peptide Activators of G-Protein-Coupled Receptors

**DOI:** 10.1021/acsomega.4c07071

**Published:** 2024-11-22

**Authors:** Yagya Prasad Paudel, Pedro A. Valiente, Jisun Kim, Philip M. Kim

**Affiliations:** 1 Donnelly Centre for Cellular and Biomolecular Research, University of Toronto, Toronto, ON M5S 3E1, Canada; 2 Department of Computer Science, University of Toronto, Toronto, ON M5S 3E1, Canada; 3 Department of Molecular Genetics, University of Toronto, Toronto, ON M5S 3E1, Canada

## Abstract

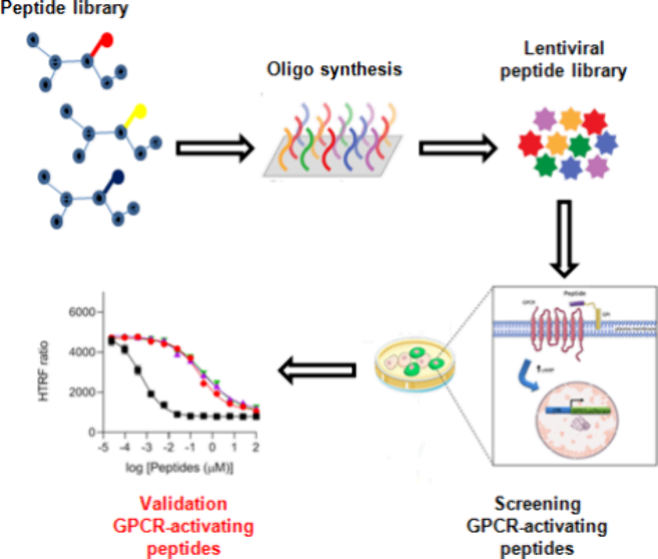

Here, we describe an innovative and efficient method for screening peptide activators of G-protein-coupled receptors (GPCRs) utilizing a protein–protein interaction (PPI) approach. We designed a library of 92,918 peptides fused with transmembrane domains of glycosylphosphatidylinositol-anchored proteins (GPI-APs). We employed a pooled lentiviral system to promote the expression of these proteins at the cellular membrane and evaluate their ability to activate GPCRs. We then used fluorescence-activated cell sorting (FACS) to screen the GPI-AP-peptide library and identify novel peptide activators of the glucagon-like peptide-1 receptor (GLP-1R). We discovered one peptide PepA3 derived from the Frizzled-like (FZ) domain of human Carboxypeptidase Z (CPZ), a regulated secreted metallocarboxypeptidase. Notably, PepA3 and its two related variants, PepA and PepA2, activated the GLP-1R receptor with less potency but comparable efficacy to that of GLP-1. We then hypothesized that all of these peptides will bind differently to the GLP-1R than the normal ligand. Our technology could identify novel GPCR-activating peptides for structure–function or drug discovery research.

## Introduction

G-Protein-coupled receptors (GPCRs) make up a large superfamily of cell surface receptors. These 7-transmembrane proteins respond to external stimuli to regulate various cellular processes, including taste, smell, vision, heart rate, blood pressure, neurotransmission, and cell growth.^1,2^ The guanine-nucleotide-binding protein family (G-proteins) is responsible for signal transmission following GPCR-agonist binding. GPCRs have become the primary pharmaceutical targets for drug discovery, covering more than 30% of FDA-approved marketed drugs due to their substantial involvement in human pathophysiology and pharmacological tractability.^3^

The GPCRs consist of three subunits called alpha, beta, and gamma and are classified into four families based on their α-subunit: Gα_i_, Gα_s_, Gα_12/13_, and Gα_q_. Most of the GPCR screening approaches rely on changes in the intracellular concentration of secondary messengers such as cyclic adenosine monophosphate (cAMP) upon ligand binding. The GPCR-dependent detection of Gα_s_, Gα_i_, and Gα_q_ helps to identify the GPCR-activating molecules because the activation increases the cAMP production.^4^ In contrast, activation of Gα_i_ inhibits cAMP production, and activation of Gα_q_ increases calcium (Ca^2+^) accumulation.^1,4^ The commonly used method for screening GPCR-activating/repressing activity is high-throughput screening (HTS) of small molecules,^5^ where many chemical compounds are screened for the activation/repression of secondary messengers. The potential hits are validated with further experiments.

Here, we present a high-throughput technology for screening GPCR-activating peptides using a PPI approach. We previously used this method to identify novel pharmacological targets.^6^ As a study example, we used fluorescence-activated cell sorting (FACS) to screen the GPI-AP-peptide library for new peptide activators of the glucagon-like peptide-1 receptor (GLP-1R). We identified one peptide PepA3 derived from the Frizzled-like (FZ) domain of human Carboxypeptidase Z (CPZ),^7^ a regulated secreted metallocarboxypeptidase. Notably, PepA3 and its two related variants, PepA and PepA2, stimulated the GLP-1R receptor with lesser potency but the same efficacy as GLP-1. We, therefore, predicted that all of these peptides would bind differently to GLP-1R than to GLP-1. Our method could help identify novel GPCR-activating peptides for structure–function or drug discovery studies.

## Results

### Designing a High-Throughput Screening Platform for Discovering Peptide Modulators of GPCRs

The lentivirus system is a versatile and efficient way to deliver expression vectors to mammalian cells.^8^ It has been successfully utilized to express GFP-tagged peptides intracellularly, paving the way to conduct screening for protein–protein inhibitors.^6^ We then used this method to display the peptides fused with transmembrane domains of glycosylphosphatidylinositol-anchored proteins (GPI-APs) on the cellular membrane and investigate their capability to activate GPCR.

We designed a peptide library comprising 92,918 unique peptides, comprehensibly covering the human secretome. It includes diverse secreted proteins, such as enzymes, cytokines, growth factors, extracellular matrix proteins, and signaling molecules. This diversity reflects the complexity of the secretome and positions the library as a powerful tool for a wide range of biological and biomedical research applications. We selected a peptide length of 32 amino acids, providing an optimal balance between representing linear and discontinuous epitopes and the practical feasibility in synthesis. This length not only ensures the inclusion of structural features like secondary motifs but also facilitates efficient and accurate synthesis using solid-phase peptide synthesis, an essential consideration for large-scale peptide production. We introduced a 14-amino-acid overlap between neighboring peptides to guarantee full sequence coverage. This overlap is crucial for ensuring complete epitope presentation, including epitopes that span the boundaries between peptides.

**Figure 1:**
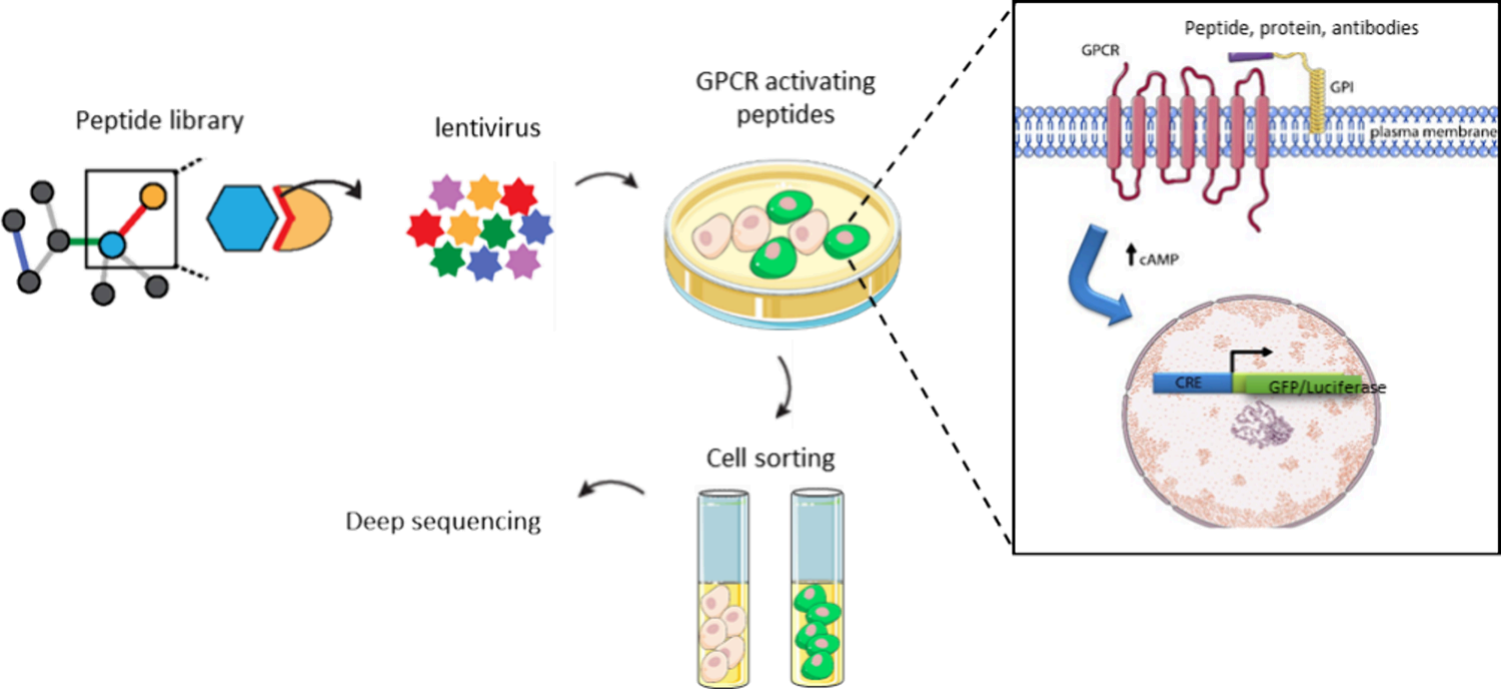
Overview of the screening platform developed to discover agonist or antagonist peptides of human GPCRs. A library of 92,918 peptides was designed, synthesized, and cloned into lentiviral vectors. We fused all of the peptides with the GPI-AP sequence to display each molecule on the cell surface. We next screened the peptide library, and the cells were sorted based on the GFP fluorescence. The positive samples were sent for deep sequencing to identify a set of candidate peptides for further experimental validation.

We then cloned these sequences into lentiviral vectors, including the GPI-AP sequence. Subsequently, cells were incubated with lentivirus-encoding GPI-AP-peptides for 24 h at a multiplicity of infection of 0.3, aiming to achieve an integration frequency of one virus particle per cell in most cases. Following that, cells were exposed to puromycin selection for 48 h to eliminate cells that were not infected. Subsequently, we screened this library to discover new peptide activators of GPCR by monitoring a GFP/luciferase reporter system regulated by a cAMP response element (CRE). When a peptide activates a receptor, cAMP levels increase intracellularly. Then, cAMP stimulates the response element binding protein (CREB), which binds to CRE and initiates the transcription of the GFP/luciferase reporter gene.^9^ The GFP/luciferase-expressing cells are next FACS sorted, their genomic DNA is extracted, and the peptide coding sequences are amplified using Illumina barcodes. Finally, these peptide coding sequences were sequenced and compared with the human genome to determine possible GPCR-activating peptides (Figure 1).

**Figure 2:**
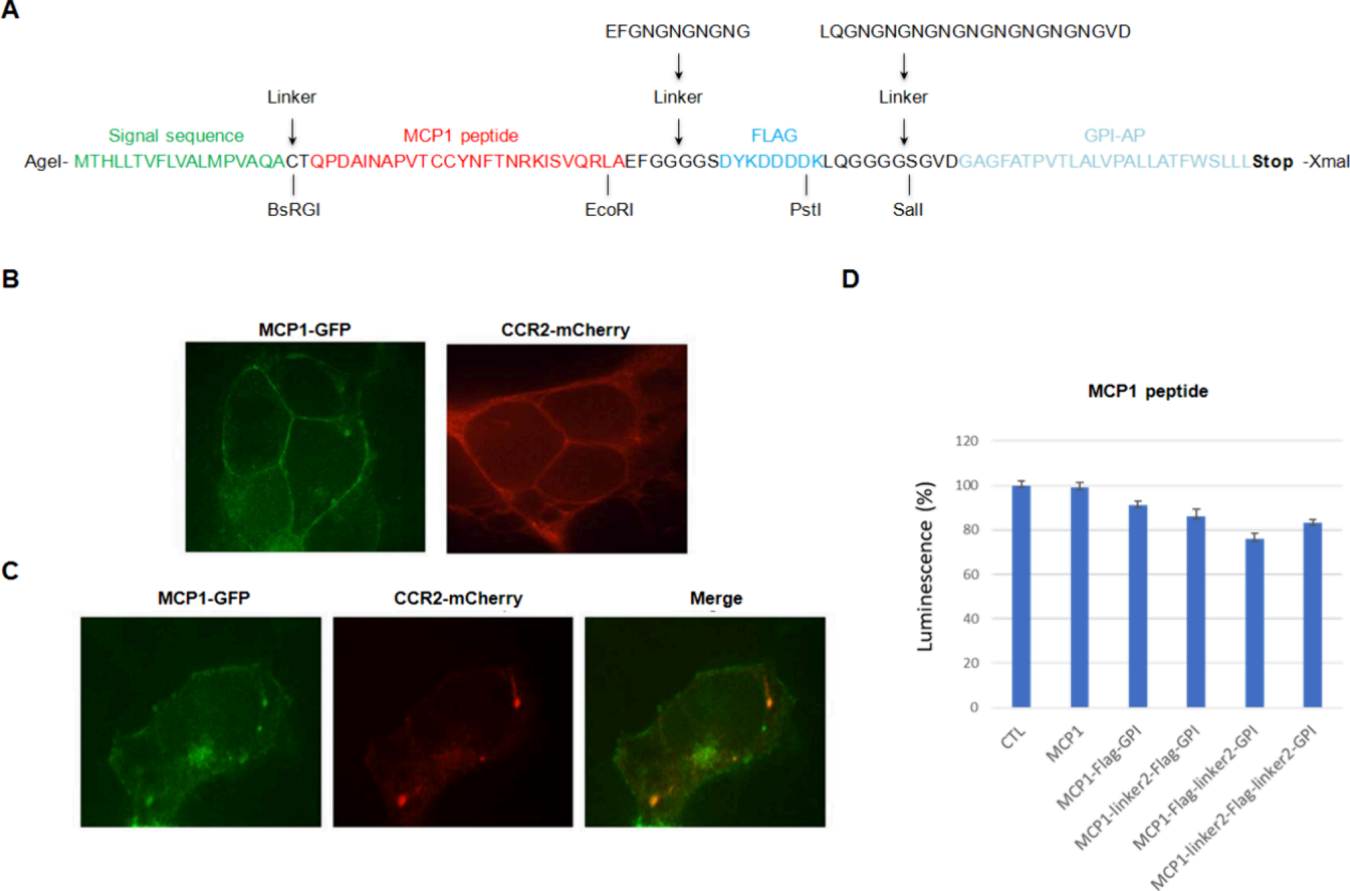
Validation of the screening platform using the MCP1 peptide. (A) Genetic construction that encodes the fusion protein used to display library peptides on the cell surface. (B) Cell surface localization of the MCP1-GFP peptide and CCR2-mCherry receptor. MCP1-GFP peptide and CCR2-mCherry were localized at the cell surface when expressed separately. (C) MCP1-GFP peptide colocalized with CCR2-mCherry at the cell surface and distinct subcellular sites upon transient coexpression in HEK293T cells. (D) Evaluating the activity of different fusion proteins of the MCP1 peptide using a CRE-luciferase reporter assay. MCP1 did not activate the CCR2 receptor when the peptide was not fused to the signal sequence and GPI-AP. The peptide activated the receptor in all the constructions where the peptide was fused to the signal sequence and GPI-AP. The activation of the different peptide variants was recorded through the inhibition of the luciferase expression induced by 10 μM forskolin.

To validate our screening platform, we chose the interaction between the chemokine MCP1 peptide and C–C chemokine receptor 2 (CCR2).^10,11^ We designed a structure that encodes a fusion protein to display the MCP1 peptide on the cell surface. The Ly6/neurotoxin (LYNX1) signal and GPI-AP sequences were present in this fusion protein’s N- and C-terminal regions, respectively. A Flag tag or GFP sequence was added upstream of the GPI-AP sequence. We also investigated the effect of various linkers between MCP1 and FLAG, as well as the FLAG and GPI-AP sequences (Figure 2A). All of the different MCP1-GFP constructions and CCR2 receptor fused with the mCherry fluorescent protein (CCR2-mCherry) were cloned into the pLJM1 nGFP lentiviral vector to evaluate their expression in human embryonic kidney 293 (HEK293) cells.

MCP1-GFP and CCR2-mCherry were localized at the cell surface when expressed separately (Figure 2B). We also observed the colocalization of MCP1 and CCR2 at the cell surface upon transient coexpression in HEK293 cells using FRET assays (Figure 2C). We then evaluated the activity of the different MCP1-GFP constructions using a CRE-luciferase reporter assay. As a negative control, we used a construction where the MCP1 peptide is not fused to the signal or GPI-AP sequences. As expected, this construction did not activate the CCR2 receptor. Notably, the MCP1 activated the CCR2 receptor in all the constructions where the peptide was fused to the signal and GPI-AP sequences (Figure 2D).

### Unveiling PepA, PepA2, and PepA3: Three Frizzled-like Derived Peptides That Act as Agonists of the GLP-1R

**Figure 3:**
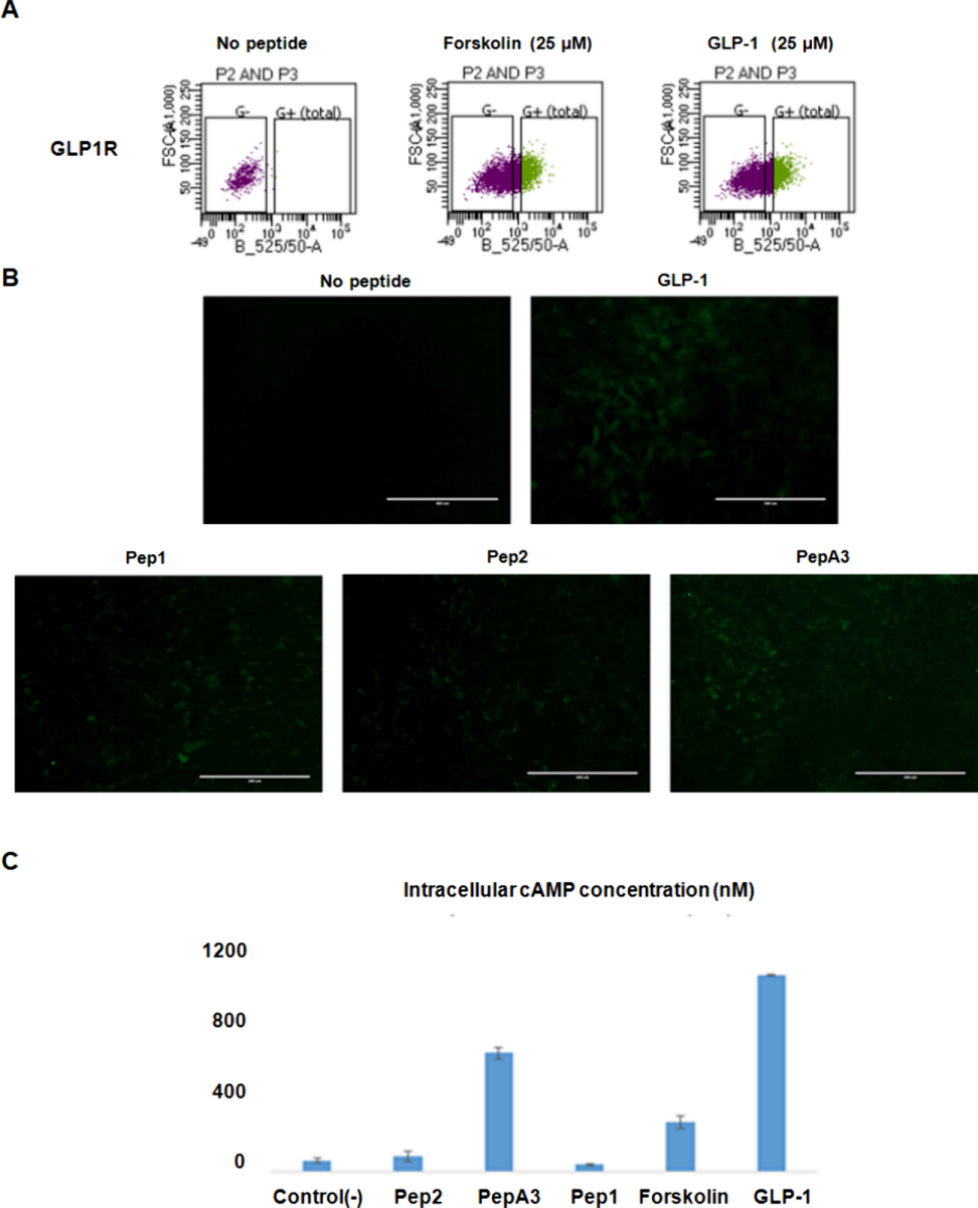
Discovery of novel agonists of the GLP-1R using our screening platform. (A) Fluorescence-activated cell analysis and sorting of HEK293 cells transfected with the GLP-1R. The left panel showed no-treatment condition, the central panel showed the treatment with forskolin, and the right is shown with GLP-1. (B) Confocal microscopy of HEK293 cells transfected with the GLP-1R treated with different peptides. (C) Evaluating the activation of the GLP-1 receptor by the identified peptides. The increase in the cyclic AMP concentration was quantified to evaluate the peptides’ activities.

We next screened the GPI-AP-peptide library using FACS to discover novel GLP-1R activators. FACS is a widely used flow cytometric technique that isolates specific cell populations from mixtures of different cells. Briefly, when a potential peptide activator stimulates GLP-1R, there is an intracellular boost of cAMP levels. Subsequently, cAMP activates CREB, which binds to CRE and triggers transcription of the GFP/luciferase reporter gene. As anticipated, we observed more GFP-positive cells in the groups treated with forskolin and GLP-1 than in the no-treatment condition (Figure 3A). We chose the top three activating peptides found throughout the screening for further validation testing (PepA3, Pep1, and Pep2). Only PepA3 stimulated the GLP-1R-expressing HEK293 cells and increased the cAMP intracellular levels (Figure 3B,C).

**Figure 4:**
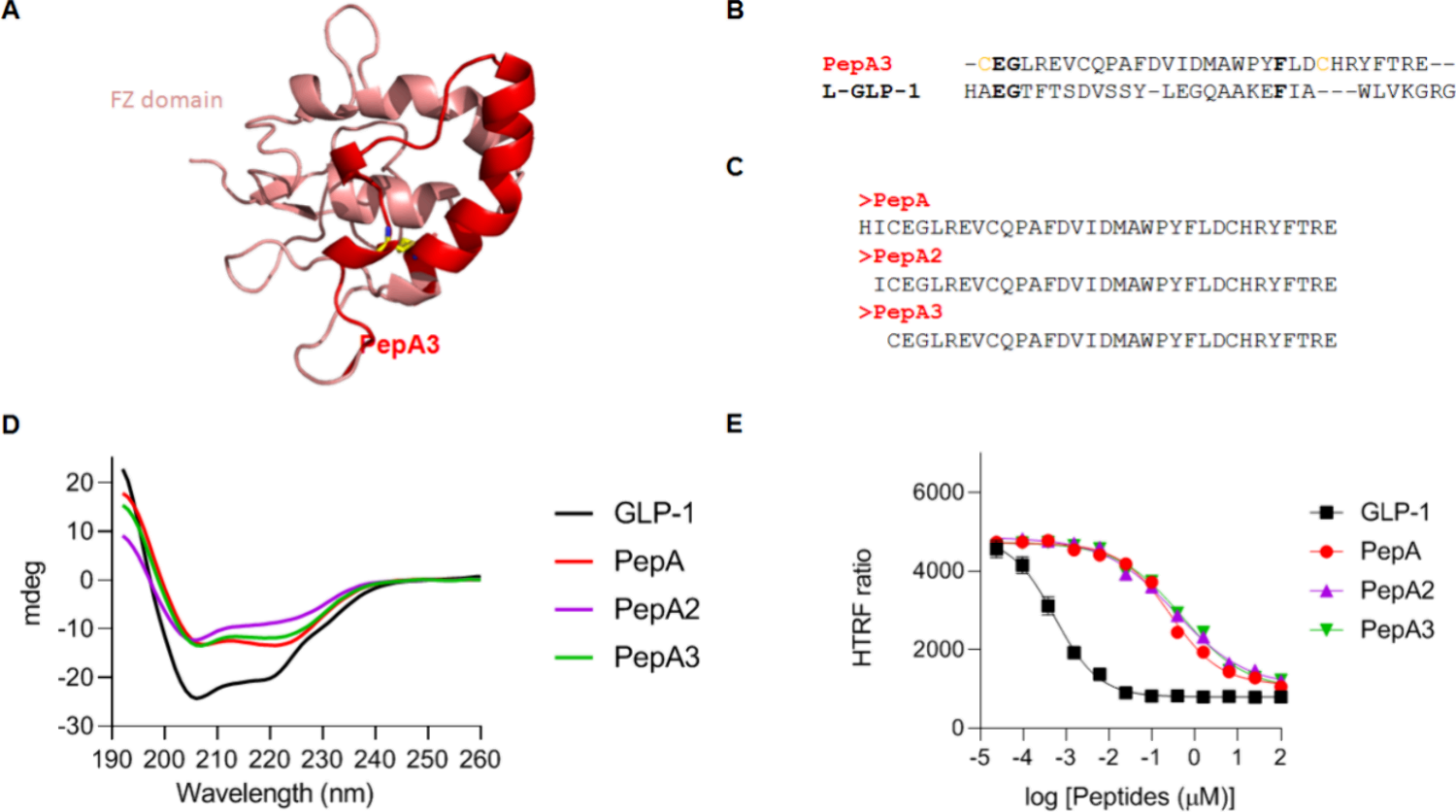
Computational analysis and experimental characterization of PepA, PepA2, and PepA3. (A) Sequence analysis identified that PepA3 (red) is within the Frizzled-like (FZ) domain (salmon) of carboxipeptidase Z. A disulfide bridge between C3 and C27 is represented as licorice (yellow, PepA numbering scheme). (B) Sequence alignment of PepA3 and GLP-1. In black bold letters are highlighted the conserved positions between both sequences. In yellow are highlighted the Cys residues within PepA3 that form a disulfide bridge. (C) Sequences of PepA, PepA2, and PepA3. (D) Circular dichroism measurements of the PepA, PepA2, PepA3, and GLP-1 in solution. All peptides were dissolved in acetonitrile:PBS (1:2). (E) Activity profiles of PepA, PepA2, and PepA3 over HEK293 cells stably expressing GLP-1R and CRE-luciferase. As a control, we evaluated the activity of GLP-1.

PepA3 is derived from the FZ domain of the human CPZ,^7^ a zinc-containing metallocarboxypeptidase that cleaves C-terminal amino acids from proteins and peptides. CPZ is secreted to the extracellular medium, and its FZ domain binds to the Wnt proteins. The 3D structure predicted for the FZ domain using Alphafold2 suggested the formation of a disulfide bridge between residues Cys3 and Cys27 of PepA3 (Figure 4A). The pair sequence alignment of PepA3 with GLP-1 indicated a low level of sequence identity between both peptides (Figure 4B). We discovered a putative trypsin cleavage site in PepA3, two residues upstream of its first residue. We then synthesized PepA3 and its related peptides, PepA2 and PepA, which have one (Ile) and two extra residues (His-Ile), respectively, for further experimental confirmation (Figure 4C). The critical role of the GLP-1 N-terminal His in the receptor activation supported our decision for adding to two extra residues in PepA3 and generating PepA,^10^ while PepA2 was designed as a control sequence.

We next performed circular dichroism (CD) analysis of the peptides in solution to determine the peptides’ secondary structure. According to our findings, PepA, PepA2, and PepA3 (30.1–46.1%) have a lower content of an α-helical structure than GLP-1 (98%) in solution (Figure 4D and Table S2). Following that, we evaluated the ability of PepA, PepA2, and PepA3 to activate the GLP-1 receptor (GLP-1R) in a stable GLP-1R-expressing HEK293 cell line. To assess the potency of GLP-1 and the discovered peptides, we used the HTRF cAMP assay. This methodology identifies intracellular cAMP by competing for the anti-cAMP antibody with d2-labeled cAMP following cell lysis. The FRET signal is then disrupted, as intracellular cAMP accumulates. GLP-1 decreased the FRET signal in GLP-1R expressing HEK293 cells with a half maximal effective concentration (EC_50_) value of 0.54 nM. Although PepA, PepA2, and PepA3 demonstrated reduced potency in decreasing the FRET signal, with EC50 values of 260, 344.9, and 488.2 nM, respectively, at higher concentrations, all three peptides were able to stimulate GLP-1R activation to a level comparable to that of GLP-1. This result suggests that while these peptides exhibit lower potency, their efficacy for activating the receptor is similar at higher concentrations (Figure 4E).

### PepA Binds Differently to the GLP-1R than the Natural Ligand

**Figure 5:**
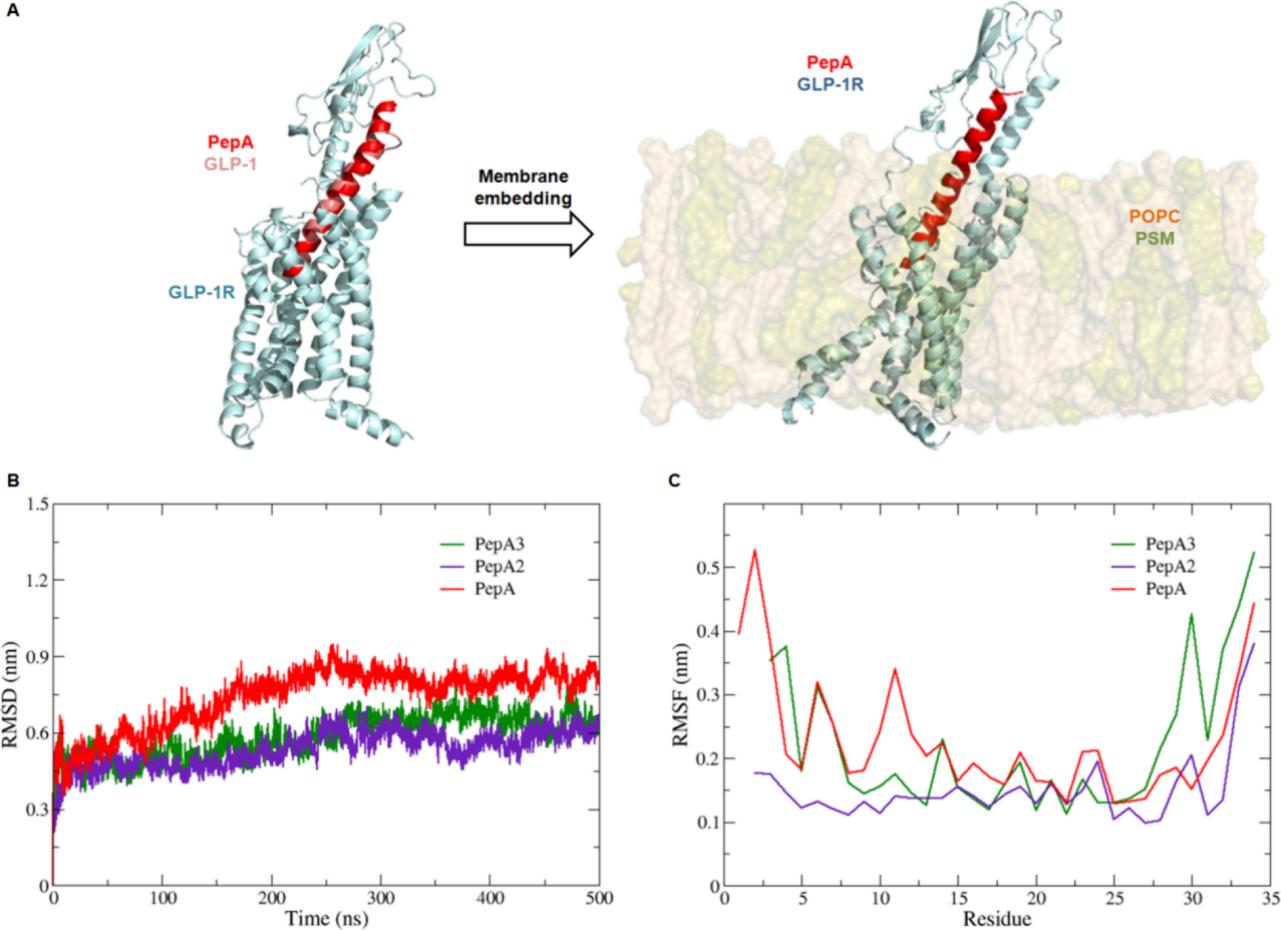
Modeling the 3D structure of PepA and its related peptides bound to the GLP-1 receptor. (A) Structural superposition of PepA (red) over the GLP-1 (salmon) structure bound to the GLP-1 receptor (GLP-1R, pdb code: 5vai). In pale cyan is shown the GLP-1R. The GLP-1R in complex with PepA and its related peptides was embedded in the POPC:PSM (1:1) membrane. The surface representation of POPC lipids is colored in orange, while that of PSM is colored in green. (B) Root-mean-square deviation (RMSD) of the heavy atoms of PepA, PepA2, and PepA3 bound to the GLP-1R. (C) Root-mean-square fluctuation (RMSF) per residue of the heavy atoms of PepA, PepA2, and PepA3 bound to the GLP-1R.

We then superimposed the PepA3 structure onto the Cryo_EM structure of GLP-1R bound to GLP-1 to build the 3D structure of GLP-1R in a complex with PepA3 (Figure 5A). The GLP-1R+PepA3 complex was embedded in a POPC:PSM (1:1) bilayer before evaluating its binding mode stability using 500 ns MD simulations. We also simulated GLP-1R bound to PepA and PepA2 (Figure 5B). All of the peptides were modeled helical without including the disulfide bridge between Cys3 and Cys27 observed in the FZ domain (Figure 4A). Significantly, all the peptides quickly stabilized in a new equilibrium position close to the initial structure, according to the RMSD profiles calculated for the peptide’s heavy atoms (Figure 5B). The calculated RMSF profiles revealed that PepA has a more extensive reorganization in the N-terminal His residue than those observed in PepA2 (Ile) and PepA3 (Cys) (Figure 5C). PepA2 showed the lowest structural fluctuation per residue among the three peptides.

**Figure 6:**
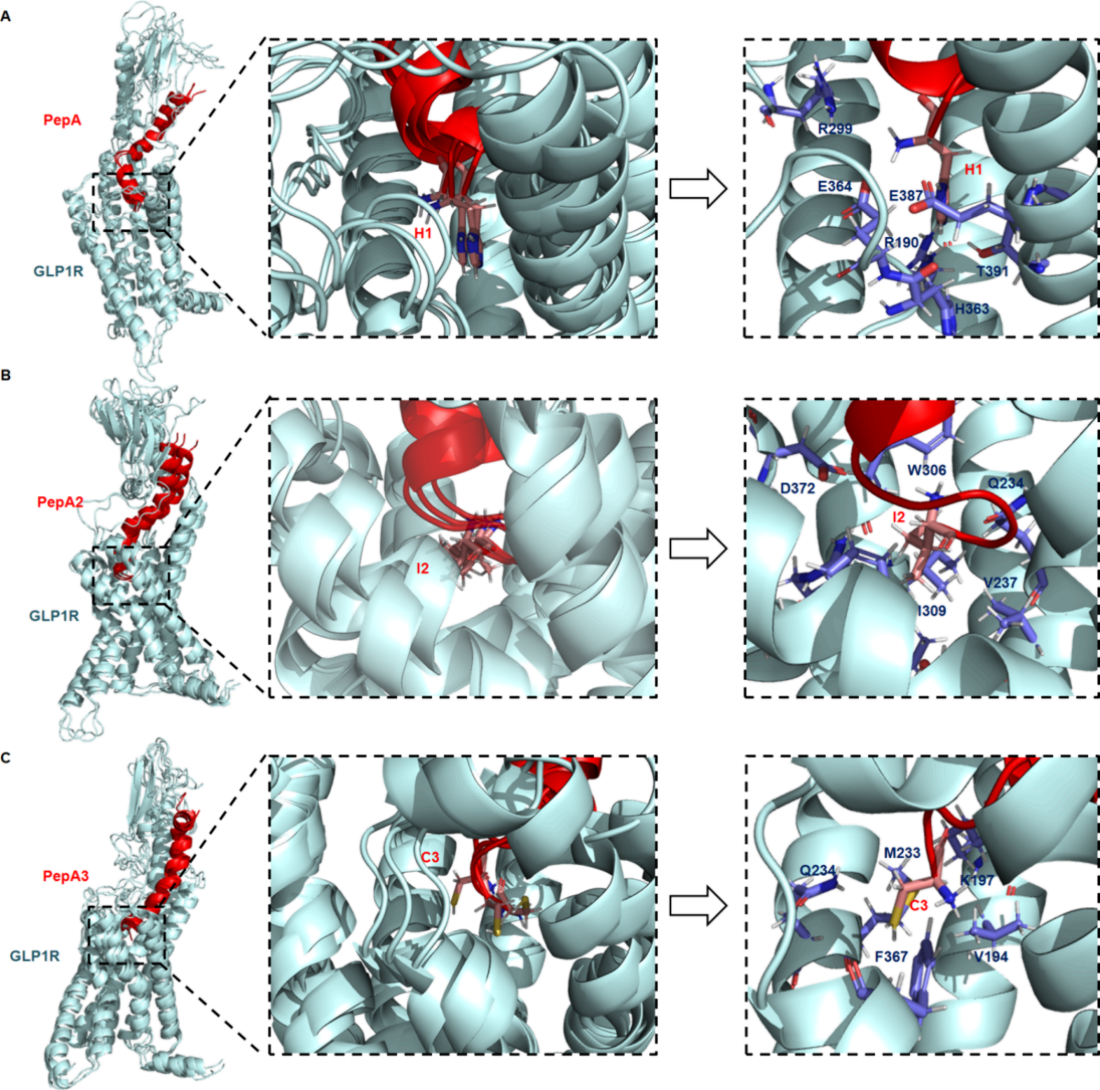
Binding mode analysis of the most representative structures extracted from the MD simulations of PepA and its related peptides bound to the GLP-1R. (A) Three most representative clusters extracted from the PepA (red) MD simulation bound to GLP-1R (pale cyan) are superimposed. The central panel zoomed in on the conformations sampled by the N-terminal residue (His1) of PepA. The right panel showed the interactions of the N-terminal residue with the receptor binding site in the most representative cluster. (B) Three most representative clusters extracted from the PepA2 (red) MD simulation bound to GLP-1R (pale cyan) are superimposed. The central panel zoomed in on the conformations sampled by the N-terminal residue (Ile2) of PepA2. The right panel showed the interactions of the N-terminal residue with the receptor binding site in the most representative cluster. (C) Three most representative clusters extracted from the PepA3 (red) MD simulation bound to GLP-1R (pale cyan) are superimposed. The central panel zoomed in on the conformations sampled by the N-terminal residue (Cys3) of PepA3. The right panel showed the interactions of the N-terminal residue with the receptor binding site in the most representative cluster.

The comparison of the three most prominent clusters obtained from the molecular dynamics (MD) simulation of GLPR1 coupled with each peptide revealed a significant level of structural similarity among these clusters (Figure 6, right and central panels). Additionally, we observed distinct interactions between each peptide’s N-terminal residue and the binding site of the GLP-1R (Figure 6, left panel).

**Figure 7:**
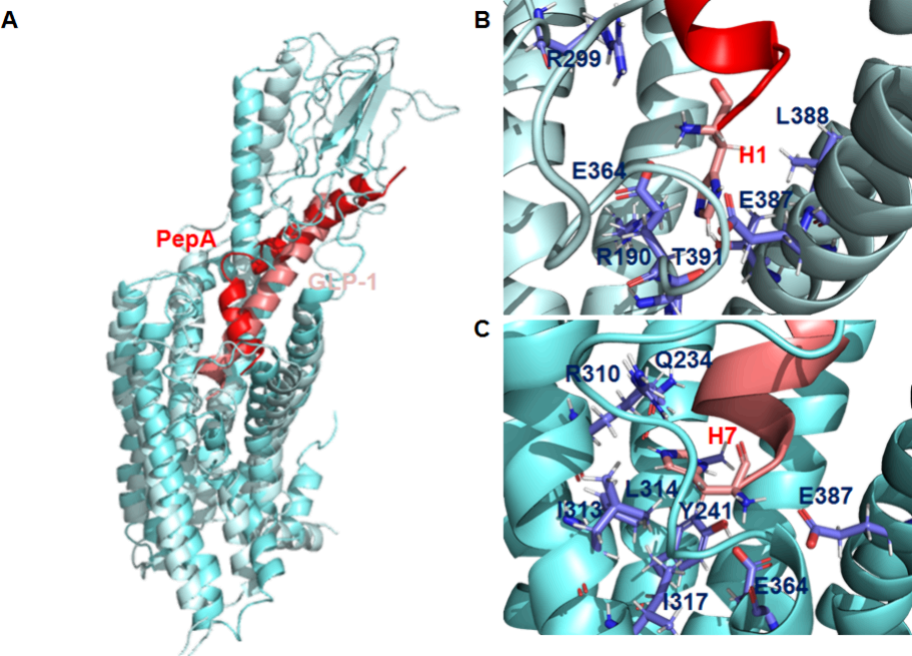
PepA binds differently to the GLP-1R than GLP-1. (A) Most representative cluster extracted from the MD simulation of PepA bound to GLPR1 was superimposed over the experimental structure of GLP-1 bound to the receptor. (B) Representation of the interactions of the PepA’s N-terminal residue with the receptor binding site in the most representative cluster. (C) Representation of the interactions of the GLP-1’s N-terminal residue with the receptor binding site in the experimental structure.

The comparison between the central structure of the most representative cluster derived from the MD simulation of GLP-1R+PepA and the experimental structure of GLP-1R+GLP-1 showed that PepA binds to GLP-1R differently than the natural ligand (Figure 7A). Despite the binding mode differences, we noticed that PepA’s N-terminal His mimics several interactions of GLP-1’s N-terminal His with the GLP-1R binding site (Figure 7B,C).

## Discussion

We present a new platform to identify previously unknown GPCR-activating peptides using a PPI approach.^6^ We found one GLP-1R-moderated activator PepA3, which is derived from the FZ domain of human CPZ, a regulated secreted metallocarboxypeptidase.^7^ PepA3 and its two related variants PepA and PepA2 stimulated the GLP-1R with lesser potency but the same efficacy as that of GLP-1. This suggests that while the peptides may not activate as strongly as GLP-1, they can still achieve similar maximum responses, indicating potential therapeutic and physiological relevance.

We hypothesize that these peptides interact with GLP-1R in a manner distinct from GLP-1, potentially providing unique opportunities for modulating receptor activity. The FZ domain of CPZ resembles the cysteine-rich domain of Frizzled receptors,^11,12^ which are involved in Wnt signaling.^13^ This structural similarity may allow it to interact with various ligands or receptors.^14^ Furthermore, the potential cleavage of this domain by ADAM7,^15^ ADAM10,^16^ or matrix metalloproteinases,^17^ known for degrading the extracellular domains of various proteins, supports the partial presence of the PepA sequence in the human secretion proteome.^18^ Despite these promising findings, further research is necessary to confirm PepA, PepA2, and PepA3 as natural alternative activators of the GLP-1 receptor. Future studies should focus on detailed structural analyses to elucidate the precise interaction mechanisms of these peptides with GLP-1R. Additionally, investigating the *in vivo* relevance and physiological effects of these peptides will be crucial to determining their potential as therapeutic agents or natural agonists.

Our screening platform offers a robust and versatile tool for discovering novel GPCR-activating peptides, particularly for orphan receptors with unknown endogenous ligands. By facilitating the identification of unique peptide activators, this approach holds significant promise for advancing structure–function studies and drug development efforts targeting GPCRs.

## Materials and Methods

### Cell Lines and Reagents

The HEK293 cell line was obtained from the American Type Culture Collection (ATCC). HEK293 cells were maintained in DMEM (Sigma) supplemented with 10% FBS and 1% penicilin/streptomycin/glutamine and the appropriate selection antibiotics when required.

### Construction of the High-Throughput Screening Platform

We designed a master library of peptides from secretome proteins. Each peptide in the library is composed of 32 amino acids. The secretome library is composed of 92,918 peptides. The lentiviral vector pLJM1 with the human CMV promoter and a green fluorescent protein (GFP) as an N-terminal tag with a puromycin selection marker was used for selecting the infected cells. The library was amplified using PCR with the oligonucleotides containing Gibson Assembly primers with BsrGI-HF and *Pst*I-HF restriction sites and purified with a gel. The pLJM1 GLP-1 plasmid was digested with BsrGI-HF and *Pst*I-HF and purified from the gel. The Gibson reaction was performed with 30 μL of HiFi DNA master mix mixed with 28 μL of plasmid (1.2 μg) and 5 μL of the PCR product (3 μg). It was incubated at 50 °C for 1 h and transformed using Endura Duos electrocompetent cells. Plasmid DNA was purified, and the library coverage, complexity, and fidelity were tested by using Sanger sequencing and Illumina deep sequencing.

The pLJM17 lentiviral vector contains the GFP gene under the control of a cAMP response element (CRE) upstream of a minimal promoter and hygromycin as the selection marker. A pLJM17 Gia Luciferase vector was generated by PCR amplification of the Gaussia Luciferase gene from the pTK GLuc (provided by the Stagljar lab) using a primer for insertion of restriction sites (*Eco*RI and *Xma*I): primer forward 5′-GGAACTAACCGGTCGCCACCATGGGAGTCAAAGTTCTGTTTGCC-3′, primer reverse 5′-CAATGCCGAATTCTTAGTCACCACCGGCCCCCTTGATC-3′. The PCR product was digested and cloned into a pLJM17 lentiviral vector.

Lentiviruses were made in a 15 cm dish format by transfecting HEK293T packaging cells with a three-plasmid system as previously described. Viral transduction was performed into HEK293T cells, stably expressing the appropriate GPCR with a multiplicity of infection of 0.3. Infected cells were selected in a puromycin-containing medium to eliminate uninfected cells, and a flow sorting methodology was employed to isolate EGFP-labeled cells.

### Validation of the Screening Platform Using the MCP1 Peptide

HEK293T cells stably expressing CCR2 and the reporter Gaussia Luciferase construct were trypsinized from subconfluent culture and seeded in a 96-well plate at a density of 5000 cells per well. Cells were incubated overnight at 37 °C in 5% CO_2_. Next, the cells were transfected with different MCP1-GFP constructions. After 6 h of incubation, 20 μL of cell medium was transferred to a black flat-bottomed 96-well plate. Next, we added 50 μL of working solution to each well containing the cell medium (Pierce Gaussia-Firefly Luciferase Dual Assay Kit, Thermo Scientific #16181). Immediately after adding the reagent, samples were read using a luminometer with a 480 nm filter.

### Fluorescence-Activated Cell Analysis and Sorting

Cells were harvested by trypsin treatment and centrifuged at 500*g* for 5 min. The pellet was resuspended in ice-cold PBS and centrifuged again. The pellet was then resuspended at a concentration of 4 × 10^6^ cells/mL in the PBS sorting buffer containing 100 Kunitz DNase I/mL, 10 μg/mL propidium iodide (Sigma), and 2% FBS. The sorting solution was also supplemented with either 10 μM forskolin, 100 μM 5,6-dichlorobenzimidazole riboside (DRB, Sigma), 10 μM forskolin (Sigma), and 100 μM DRB or DMSO, as a control. The cells were then sent through a 40 μm filter to remove large clumps and loaded into either a FACScan Flow Cytometer (BD Bioscience) for cell analysis or a FACSVantage SE cell sorter (BD Bioscience) for cell sorting. The cells with positive propidium iodide staining, i.e., the dead cells, were first eliminated from the analysis or sorting pool. For cell sorting, the desired population, either the EGFP brightest or least bright ones according to the purpose of experiments (see Results), was sorted into either 15 mL conical tubes or 96-well plates, which contained complete DMEM culture media.

### Genomic DNA Preparation and Illumina Sample Preparation

Genomic DNA (gDNA) from peptide-expressing cells at different time points was extracted using a QIAamp DNA Blood Mini Kit. PCR amplification of peptides from gDNA in parallel with the lentiviral plasmid library (naive library) was performed using indexed Illumina PCR primers to incorporate the Illumina adapter and indexing sequences. Each 50 μL reaction mixture contained 3.2 μg of template, 2× PCR buffer, 2× enhancer solution, 300 μM each dNTP, 900 nM each for Adapter A (5′-AATGATACGGCGACCACCGAAATGGACTATCATATGCTTACCGTAACTTGAA-3′), and Adapter B (5′-CAAGCAGAA-GACGGCATACGATGTGGATGAATACTGCCATTTGTCTCGAGGTC-3′), 1 mM MgSO4, 3.75 units of Platinum Pfx polymerase, and water to 50 μL. The PCR reaction was performed by denaturing at 94 °C for 5 min, followed by (94 °C for 30 s, 65 °C for 30 s, and 68 °C for 30 s) ×28 and 68 °C for 5 min, then cooling to 4 °C. The resulting 244 bp product was purified by electrophoresis in 2% agarose followed by gel extraction. Peptide libraries were quantified using a Quant-It assay (Invitrogen) and pooled. The inset size of the pooled library was confirmed on an Agilent Bioanalyzer High Sensitivity DNA chip (Agilent Technologies), and the size-corrected concentration was determined with RT-qPCR (KAPA biosystems Illumina standards). Peptide library (11.4 pM) and 0.6 pM PhiX control library (Illumina) were denatured and loaded on a HiSeq 2000 V3 150 cycle sequencing kit, with a read length of 150 bp.

### cAMP Assay for Validating GLP-1R Activating Peptides

The stable cell lines were prepared by the infection of HEK293T cells with the pLJM1 vector with GLP-1R and CRE. The cells were selected with the hygromycin antibiotic. Stable cell lines were transfected with the construct expressing GLP-1 and Pep1, Pep2 and PepA3 or the pcDNA3.1 empty vector using PolyJet Transfection Reagent (FroggaBio) and cultured for 24 h. The culture medium was changed, and cells were grown for 24 h. The cAMP cell content was estimated using a homogeneous time-resolved fluorescence resonance energy transfer (TR-FRET) immunoassay using a cAMP-Gs Dynamic kit (PerkinElmer) following manufacturer’s protocol.

### Peptide Synthesis

All peptides were synthesized, purified, and characterized by Lifetein LLC. All peptides’ purity is higher than 90%. In the Supporting Information material, we provided the details about the characterization of these peptides (molecular weight, purity, HPLC, and MS) (Figures S1–S4 and Table S1).

### Circular Dichroism (CD) Analysis

Peptide secondary structure determination was carried out using a Jasco J-720 spectropolarimeter (Table S2). Lyophilized peptide powders were dissolved in acetonitrile:PBS (1:2), and CD spectra were read immediately. Peptide concentrations were 20 μM for GLP-1, PepA, PepA2, and PepA3 in acetonitrile:PBS (1:2). Samples were read using a 0.1 cm cuvette path length with 10 accumulations per run and 50 nm/min scanning speed. All spectra were background subtracted.

### HTRF cAMP Assay

cAMP accumulation was measured using a HTRF cAMP-Gs Dynamic kit (Cisbio Bioassays, 62AM4PEB) according to the manufacturer’s instructions. Briefly, HEK293 cells expressing hGLP1R were trypsinized from subconfluent culture and seeded in a 96-well low-volume plate at a density of 2000 cells per well. Cells were treated with different concentrations of GLP-1, PepA, PepA2, or PepA3 peptides. After 4 h of incubation at 37 °C, cAMP d2 reagent and cAMP Eu-Cryptate antibodies were added to each well. After incubation at room temperature for 30 min in the dark, the plate was read using a Synergy 2 plate reader (BioTek) with excitation at 330 nm and emission at 620 and 665 nm. Data were used to calculate the EC_50_ value by fitting it to a nonlinear sigmoidal curve using GraphPad Prism 8.

### Molecular Dynamics Simulations

All peptide 3D structure was modeled and superimposed onto the Cryo_EM structure of GLP-1R bound to GLP-1 to build the 3D structure of the GLP-1R+peptide complexes using Modeler^19^ version 9.14. All the initial structures and topology files for the MD simulations of the GLP-1 receptor (GLP-1R) in complex with different peptides embedded into a POPC:PSM (1:1) bilayer were built using the membrane builder generator implemented in the CHARMM-GUI web server.^20^ The GROMACS software package^21^ version 2019.3 was used to perform the molecular dynamics (MD) simulations of the GLP-1R+peptide complexes using the CHARMM36-m force field^22^ and TIP3P water model.^23^ Two consecutive energy minimization (EM) schemes were used to relax the systems initially. The systems were then equilibrated in two sequential NVT ensemble simulations before being equilibrated in five successive NPT ensemble simulations at *p* = 1 bar and *T* = 310 K. We gradually released the position restraints applied to the protein-heavy atoms in both steps. Finally, the production NPT runs were performed for 300 ns for each system.

### Statistical Analysis

Statistical significance was analyzed by a two-tailed unpaired Student’s *t* test using MS Excel. A *P* value of less than 0.05 was considered to be statistically significant.

## Supporting Information Available

Sequences of the peptides evaluated experimentally in this study; estimated secondary structure content (%) of GLP-1, PepA, PepA2, and PepA3; analytical characterization of the PepA peptide provided by the Lifetein company; analytical characterization of the PepA2 peptide provided by the Lifetein company; analytical characterization of the PepA3 peptide provided by the Lifetein company; and analytical characterization of the GLP-1 peptide provided by the Lifetein company (PDF)

## Abbreviations

**cAMP**: cyclic adenosine monophosphate
**CD**: circular dichroism
**DMEM**: Dulbecco’s modified Eagle’s medium
**DMSO**: dimethyl sulfoxide
**GLP-1**: glucagon-like peptide-1
**EM**: energy minimization
**GLP-1**: glucagon-like peptide-1
**GLP-1R**: glucagon-like peptide-1 receptor
**GPCR-Gs**: G-protein-coupled receptors
**HPLC**: high-pressure liquid chromatography
**HTRF**: homogeneous time-resolved fluorescence
**EC**_**50**_: half maximal effective concentration
**MD**: molecular dynamics
**MS**: mass spectra
**PBS**: phosphate-buffered saline
**PDB**: protein data bank
**PSM**: palmitoylsphingomyelin
**POPC**: 1-palmitoyl-2-oleoylphosphatidylcholine, palmitoyloleoylphosphatidylcholine
**RMSD**: root-mean-square deviation
**RMSF**: root-mean-square fluctuation
