# Supplementary material for: A High-Throughput Method for Screening Peptide Activators of G-Protein-Coupled Receptors
Source: ACS Omega. 2024 Nov 22;9(49):48471–9. doi: 10.1021/acsomega.4c07071 (PMC11635519; doi:10.1021/acsomega.4c07071)
Supplement: Supplementary file 1 — ao4c07071_si_001.pdf [file ao4c07071_si_001.pdf]

Supporting Information

A High Throughput Method for Screening Peptide Activators of G-protein-coupled receptors

Yagya Prasad Paudel [1+], Pedro A Valiente[1+], Jisun Kim[1+], and Philip M Kim[1, 2, 3*]

[1] Donnelly Centre for Cellular and Biomolecular Research, University of Toronto, Toronto, ON M5S 3E1, Canada.
[2] Department of Computer Science, University of Toronto, Toronto, ON M5S 3E1, Canada.
[3] Department of Molecular Genetics, University of Toronto, Toronto, ON M5S 3E1, Canada.

[+] These authors contribute equally

Corresponding author

*email: pm.kim@utoronto.ca

Contents:

Table S1 Sequences of the peptides evaluated experimentally in this study.

Table S2 Estimated secondary structure content (%) of GLP-1, PepA, PepA2 and PepA3 by BeStSel.

Figure S1 Analytical characterization of the PepA peptide provided by the LifeTein company. A) HPLC report. **B)** Mass spectrometry report.

Figure S2 Analytical characterization of the PepA2 peptide provided by the LifeTein company. A) HPLC report. **B)** Mass spectrometry report.

Figure S3 Analytical characterization of the PepA3 peptide provided by the LifeTein company. A) HPLC report. **B)** Mass spectrometry report.

Figure S4 Analytical characterization of the GLP-1 peptide provided by the LifeTein company. A) HPLC report. **B)** Mass spectrometry report.

Table S1 Sequences of the peptides evaluated experimentally in this study

Peptides	Full sequence[a]	Purity (%)	MW (g/mol)
**GLP-1**	HAEGTFTSDVSSYLEGQAAKEFIAWLVKGRG	96.80	3355.80
**PepA**	HICEGLREVCQPAFDVIDMAWPYFLDCHRYFTRE	90.15	4133.73
**PepA2**	ICEGLREVCQPAFDVIDMAWPYFLDCHRYFTRE	90.55	3996.52
**PepA3**	CEGLREVCQPAFDVIDMAWPYFLDCHRYFTRE	94.61	3883.37
**Pep1**	ELAELVPRLFRVASKTHRDLLEVNLQNGILFV	93.49	3689.06
**Pep2**	SGLAGHSVFLVPGLAAHPGSHSDQPPGVPSRR	95.55	3188.58

Table S2 Estimated secondary structure content (%) of GLP-1, PepA, PepA2 and PepA3 by BeStSel.

Peptide	α-helix	β-sheet	Others
**GLP-1**	98.5	1.5	0
**PepA**	46.1	8.5	45.4
**PepA2**	30.1	13.3	56.5
**PepA3**	42.9	10.4	46.7

A

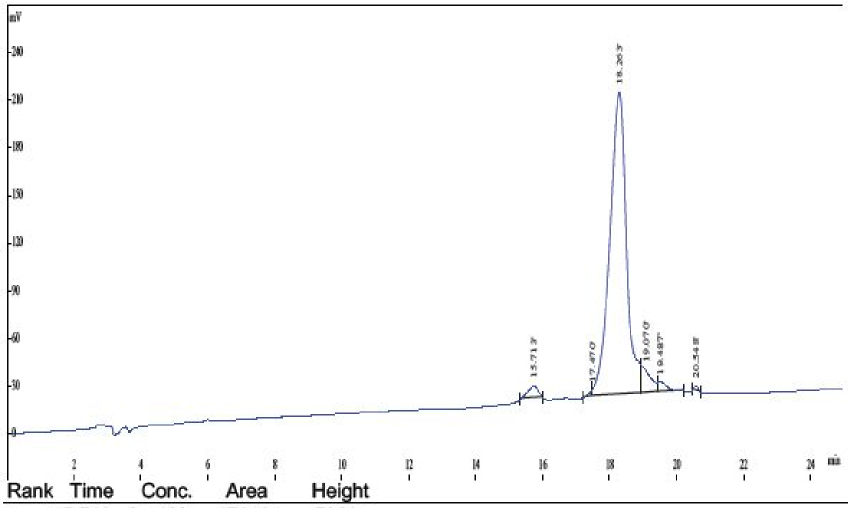

Rank	Time	Conc.	Area	Height
1	15.713	2.4463	178134	7923
2	17.470	0.4882	35553	5195
3	18.263	90.1578	6565086	190829
4	19.070	4.6990	342174	14000
5	19.487	1.7823	129786	6408
6	20.548	0.4264	31050	3501
Total		100	7281783	227856

B

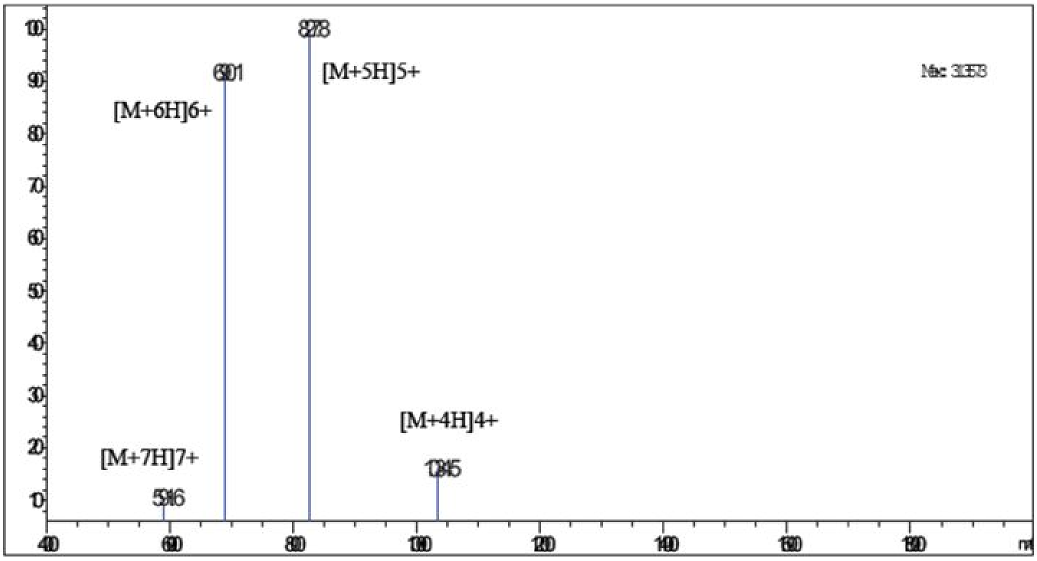

Sample:866908 **MW: 4133.73**

Figure S1 Analytical characterization of the PepA peptide provided by the LifeTein company. A) HPLC report. **B)** Mass spectrometry report.

A

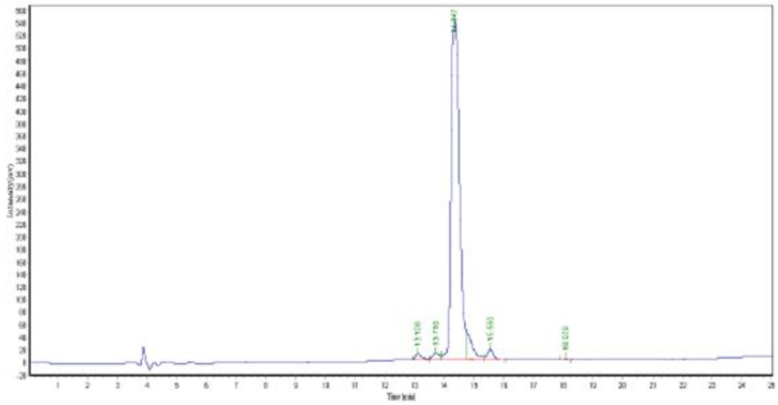

Peak No.	Ret Time	Height	Area	Conc..
1	13.108	9666.405	135858.922	1.1602
2	13.710	10782.307	178617.625	1.5254
3	14.347	539540.313	10603939.000	90.5574
4	14.347	42516.199	553782.750	4.7293
5	15.550	16523.646	224029.797	1.9132
6	18.078	1434.529	13408.003	0.1145
Total			100.0000	

B

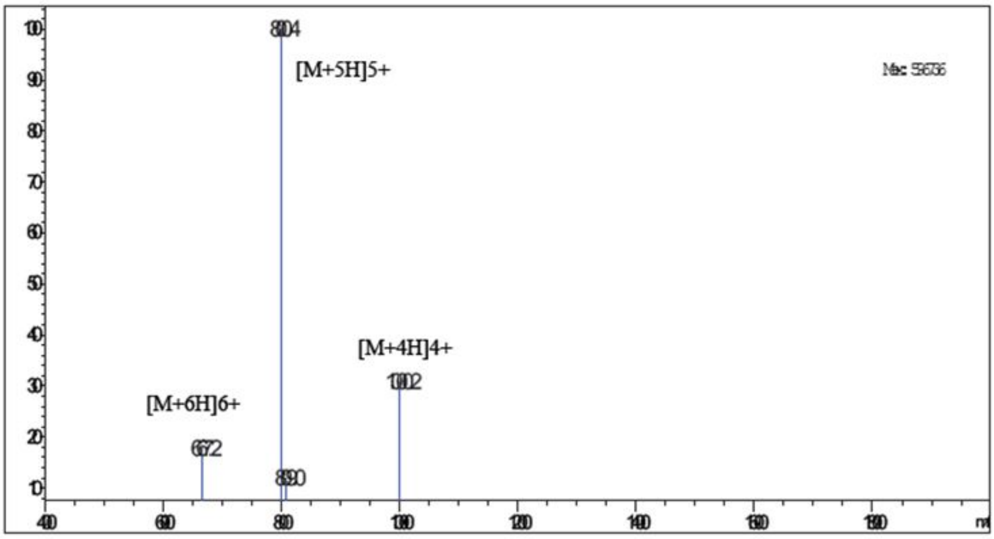

Sample: 869136 **MW: 3996.52**

Figure S2 Analytical characterization of the PepA2 peptide provided by the LifeTein company. A) HPLC report. **B)** Mass spectrometry report.

A

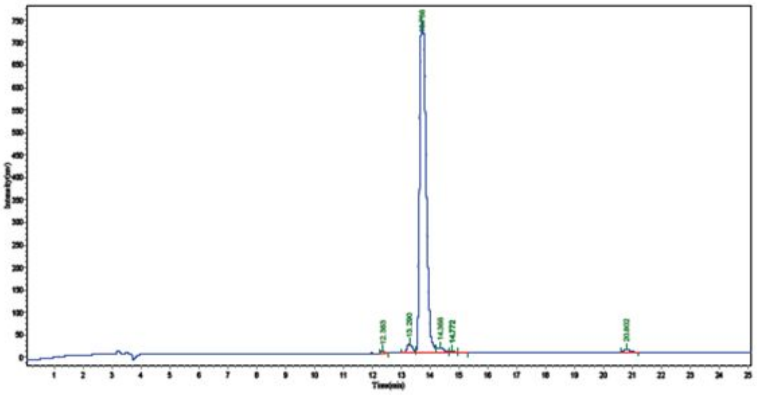

Peak No.	Ret Time	Height	Area	Conc..
1	12.383	4050.844	36582.395	0.2921
2	13.290	19324.064	269158.188	2.1493
3	13.738	738203.500	11848154.000	94.6114
4	14.355	11062.903	187271.484	1.4954
5	14.772	4539.445	54986.336	0.4391
6	14.772	1747.900	17144.107	0.1369
7	20.802	6609.769	109673.320	0.8758
Total			100.0000	

B

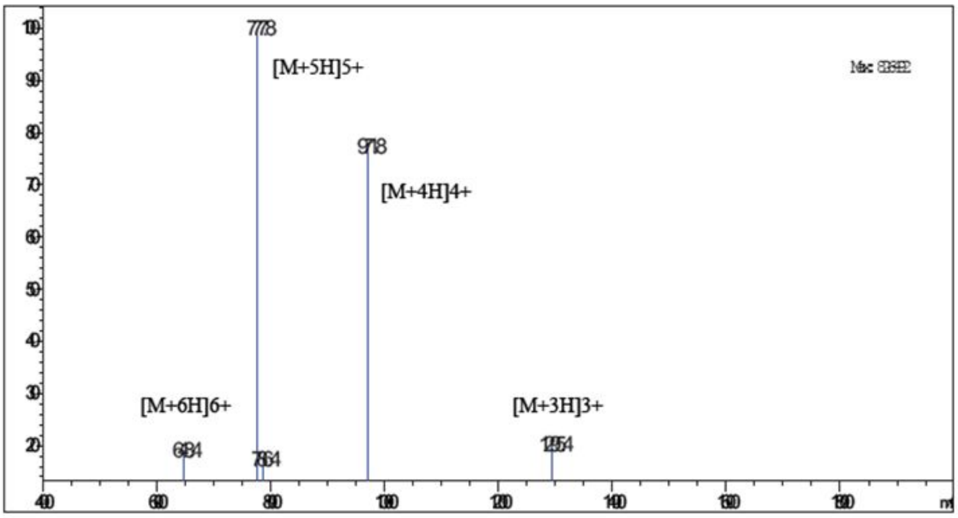

Sample: 869137 **MW: 3883.37**

Figure S3 Analytical characterization of the PepA3 peptide provided by the LifeTein company. A) HPLC report. **B)** Mass spectrometry report.

A

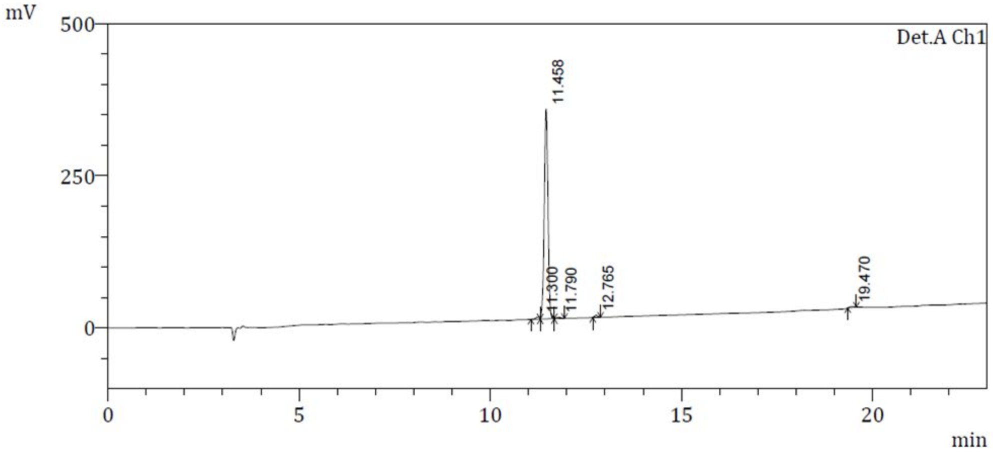

B

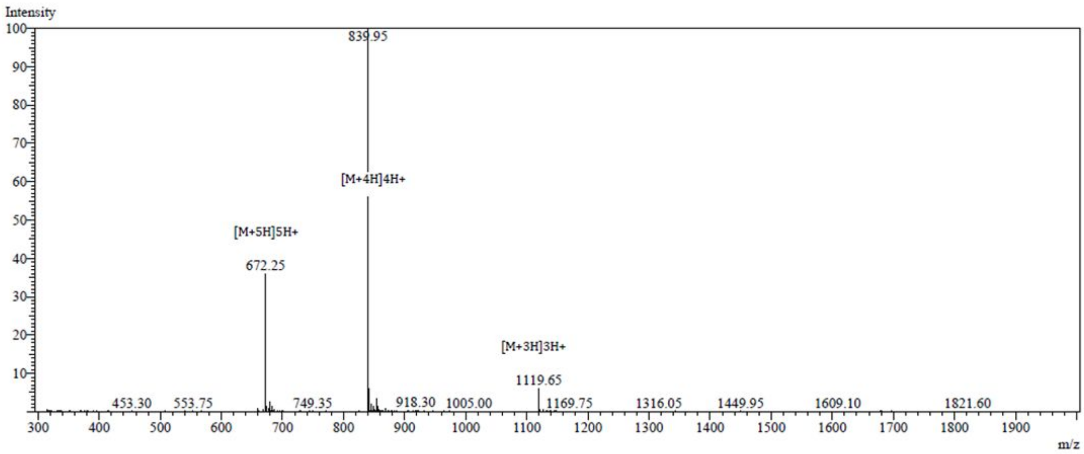

Figure S4 Analytical characterization of the GLP-1 peptide provided by the LifeTein company. A) HPLC report. **B)** Mass spectrometry report.
